# Achilles' tendon rupture dancing the ‘Jerusalema’ – A case series

**DOI:** 10.1016/j.ijscr.2021.106368

**Published:** 2021-09-07

**Authors:** Ryan Roopnarinesingh, Robert Kenyon, Luke Turley, Aoife Feeley, Thomas Bayer, Khalid Merghani

**Affiliations:** Midland Regional Hospital Tullamore, Orthopaedic Department, Tullamore, Co. Offaly R35 NY51, Ireland

**Keywords:** Achilles tendon rupture, Jerusalema, Viral dance video, Accelerated rehabilitation programme, Early weightbearing, Surgery, Trauma and orthopaedics

## Abstract

**Introduction and importance:**

The weekend warrior has long been prey to musculoskeletal injuries as a result of intermittent, high intensity activity. The Achilles tendon is known to be particularly vulnerable in this population cohort but during the COVID-19 lockdowns in Ireland and all over the world there has been a certain level of detraining and deconditioning among all age groups and populations.

Throughout the worldwide restrictions, viral internet challenges and dances have encapsulated the spirit of a global community with the ‘Jerusalema’ dance being no exception. The rise of this particular viral sensation was at the detriment of the Achilles tendons of three middle aged gentlemen on who we base our case series.

**Presentation of cases:**

Over the space of ten days three cases of Achilles tendon rupture repair presented to the emergency department in Midlands Regional Hospital Tullamore (MRHT) with the mechanism of tendon rupture being through the ‘Jerusalema’ dance.

These patients were surgically managed in line with local institution practice and postoperative outcomes were good with no complications noted. Follow up is ongoing.

**Clinical discussion:**

This retrospective case series is based on the impact of the ‘Jerusalema Dance’ on presentations of Achilles tendon rupture to the Emergency Department in a single regional hospital from January to March 2021. We used these cases in conjunction with a review of current literature to highlight the benefit of an integrated Achilles Tendon rehabilitation programme in this at-risk patient cohort.

**Conclusion:**

This paper highlights the dangers inherent when well intentioned, but physically deconditioned individuals endeavour to perform a physical exercise which is deceptively demanding. Going forward, viral challenges such as the ‘Jerusalema’ may contribute to new and interesting mechanisms of injuries in our ‘weekend warrior’ cohort. In addition to this, given the global deconditioning seen due to the COVID 19 pandemic and subsequent lockdowns we may see a higher rate of Achilles tendon injuries in the near future across a multitude of patient cohorts. Level one evidence suggests that conservative treatment is just as effective as surgical treatments in the majority of patients with an Achilles tendon rupture, as long as a protocol of rehabilitation with early weightbearing is performed. Our accelerated rehabilitation programme in MRHT is in line with others however internal audit and new literature in the future may enable us to refine it further.

## Highlights

• Achilles Tendon Rupture is common amongst the conditioned and deconditioned population.
• Surgical and conservative management options are widely explored in the literature.
• Viral dance challenges such as the Jerusalema offer new and interesting patterns of injury.
• An early weightbearing rehabilitation programme after TA rupture shows lower re-rupture rates.

## 1 Introduction

While the Jerusalema song originated from South Africa, the dance stems from Angola where a dance group recorded the steps to the song while eating their lunch. Since then, it has become a viral internet sensation with dance groups, charities, police and healthcare workers all getting involved posting videos to social media. The movements themselves include small jumps with frequent foot switches and occasional push offs. It would likely be described as a low intensity exercise, however given the rapid weight transfer required, and the moderate complexity of the dance sequence overall, it may prove relatively demanding to a deconditioned participant.

The Achilles Tendon (TA) endures a significant amount of strain and is at risk of rupture in all age groups upon running, jumping and exercises requiring sudden changes in velocity.

Multiple epidemiological studies have shown that the overall incidence of TA ruptures is on the rise globally [1], [2], [3]. This may be attributed to in part by our aging population, sedentary lifestyle and prevalent co-morbidities such as diabetes, hypertension and obesity [4].

Upon rupturing the TA, patients commonly report the feeling of a ‘pop’ or as if someone had kicked them in the back of the leg. Pain may or may not be present.

More objective assessments upon presentation to hospital include weakened or absent plantar flexion when standing, a palpable gap in the TA and a positive Thompsons test or Matles test [4], [5].

Some debate still remains as to whether acute Achilles tendon ruptures are best treated conservatively or operatively. Many randomized controlled trials and meta-analysis have failed to show a statistically significant difference between operative and non-operative management [6], [7], [8].

While early operative approaches confer potentially greater stability and lower re-rupture rates, non-operative management gives similar long term outcomes while mitigating the risk of post-operative infection [9].

Regardless of operative or non-operative treatment the rehabilitative phase of TA rupture has seen a shift towards early weight bearing and graduated physiotherapy. There is extensive research showing it is safe, has a low complication rate and achieves a superior functional outcome to conventional immobilisation [7], [10], [11], [12], [13].There is however a lack of consensus on what exactly these functional rehabilitation programmes should consist of given that most trials to are heterogenous in their outcome measurements [14].

Through this case series we hope to highlight one high volume Irish trauma centres current protocols with regards to Achilles tendon rupture management and highlight some of the new literature available to us on the topic.

## 2 Cases

Over the space of ten days all three of these cases presented to the emergency department in Midlands Regional Hospital Tullamore (MRHT).

### 2.1 Case 1

The first case is of a fifty year old gentleman who presented to the Emergency department with weakness and pain of his left lower limb following dancing the ‘Jerusalema’ with his granddaughter for a charity video.

He described feeling like someone had ‘kicked the back of his leg’ as he pushed off during one of the movements and subsequently was unable to push off the ground to walk (Pictures 1–4). On clinical examination, Thompson Test was positive and a palpable tendinous gap was felt approximately 5 cm from the calcaneal insertion.

**Pictures 1–4:**
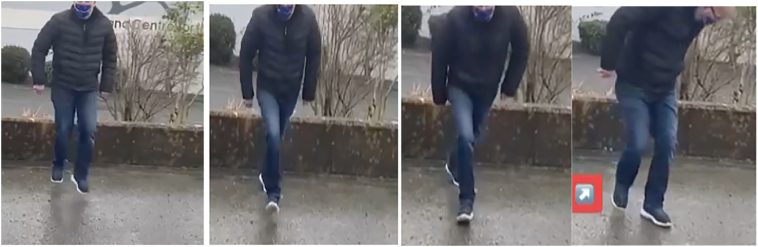
Sequence of events leading to TA rupture (Pictures with patients consent).

His past medical history was significant for Rheumatoid arthritis, well controlled Epilepsy and he was a smoker of approximately 15 cigarettes a day.

His medications included Methotrexate and Sodium Valproate.

### 2.2 Case 2

The second case is of a fifty-eight year old gentleman who had been dancing the ‘Jerusalema’ with his granddaughter and felt a pop at the back of his right leg. This was followed by severe pain and weakness on plantarflexion. He presented to the emergency department in MRHT one day later and on clinical examination was Thompson test positive and had a palpable tendinous gap.

He had a multimorbid history of Ischaemic Heart Disease with prior Percutaneous Coronary Stenting in 2006. He is on Aspirin for the same. He also suffers from Hypertension, Diverticular Disease and had a Microdiscectomy in 2019. He is an ex-smoker.

### 2.3 Case 3

The third case was of a forty two year old gentleman who again had been dancing the ‘Jerusalema’ with his daughter at home and felt a sudden pop at the back of his left leg. He described the injury occurring in a similar fashion to our last patient following a small jump and push off the floor with his left leg.

**Figure:**
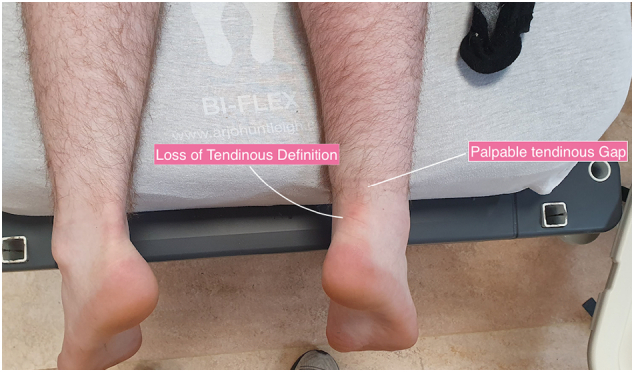

He presented to the emergency department in MRHT where he was clinically diagnosed with a ruptured Right TA with a clear loss of tendinous definition and a palpable tendinous gap approximately 4–6 cm from its calcaneal insertion (Picture 1). His past medical history was unremarkable except for an elevated Body Mass Index (BMI) of 32 and he is a non-smoker.

All three patients recalled the injury occurring at a specific part of the dance with one gentleman having caught it on video (Pictures 1–4).

Of note none of our patients had recently taken any class of antibiotics or steroids prior to their injury.

Upon review, all three gentlemen were offered either an operative or a non-operative management approach and were counselled on the risks and potential benefits of each. All three of them opted for an operative approach.

Each one of our three patients was operated on the day after presentation to the emergency department on a scheduled trauma list.

In all three cases they were given a general anaesthetic, prophylactic antibiotics in accordance with local guidelines and were positioned prone for the operation.

The TA was approached via a 5-7 cm incision medial to the tendon with care taken to avoid damage to the soft-tissue envelope by utilizing only gentle traction of the skin edges for exposure.

Intra operatively all three gentlemen were seen to have a complete TA ruptures.

Each patient underwent an open end to end modified Krakow repair of the tendon with a 5 Ethibond suture and 2/0 Vicryl circumferential suture with the foot in plantar flexion. The posterior compartment was decompressed in all 3 cases. The contralateral extremity was used as a guide for the restoration of proper tendon length.

The subcutaneous tissue was closed with 3/0 Vicryl interrupted sutures and the skin was closed with 3/0 Quill. The wounds were dressed in the operating theatre and all three patients were put in a walking boot with a one inch heel raise putting them in approximately 20 degrees of plantar flexion.

There were no intraoperative complications in any of the surgeries.

All patients were given one more dose of intravenous antibiotics on the ward, analgesia as required and were also placed in an Unna compression bandage prior to discharge on post-operative day one.

They were educated on the walking boot, weightbearing status, symptoms of lower limb venous thrombosis and were given follow up dressings clinic and outpatients clinic appointments.

All three gentlemen were seen back in our outpatients dressing at 12 days for wound review of which there were no post-operative wound complications. Subsequently at their outpatients clinic review at three weeks post-operative, all patients were clinically well. The one inch heel raise was removed from the walker boot. Weightbearing instructions were given, and controlled ankle range of movement exercises limiting dorsiflexion to neutral was employed.

Subsequent outpatient clinic visits have been uneventful with all three gentlemen progressing with no issues. They are currently in phase 3 of our accelerated rehabilitation programme (see Discussion) and are attending the outpatient physiotherapy clinic for gait retraining.

## 3 Discussion

The Achilles Tendon is able to withstand between 2000 and 9000 Newtons of stress depending on the applied load [15]. It is known that there is a natural aging effect that afflicts tendons with mean collagen diameter and content decrease resulting in an overall decline in mechanical properties of the tendon. With tendons being a viscoelastic substance that exhibits stress-strain behaviour, in a subject that is deconditioned, their lack of regular exercise may make them more prone to both fatigue failure with repetitive microtraumas to the tendon or macrotrauma due to a load applied above the tensile strength of the tendon [16].

The Achilles itself is prone to an acute rupture at its hypovascular area, 4-6 cm proximal to its calcaneal insertion and is the site of rupture in all three of our case reports.

**Figure:**
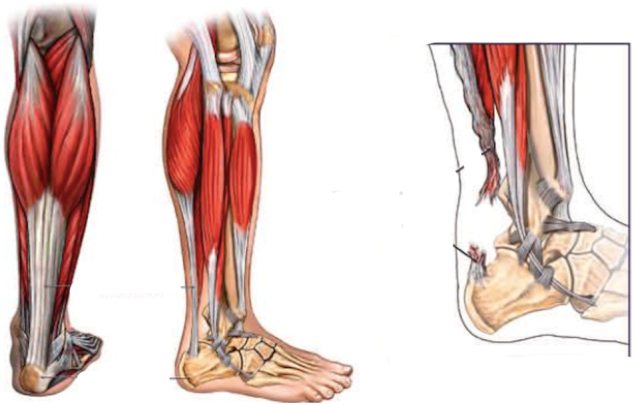

On reviewing the Jerusalema dance, there is a push off sequence in which the participant accelerates forward while simultaneously rapidly transferring weight to the alternate foot which is prepositioned in plantarflexion. The gastrocsoleus complex is pre-tensioned prior to the foot making contact with the ground. This is further exacerbated by the knee being in an extended position which increases the tension through the gastrocnemius.

Sudden loading of the forefoot in this position can momentarily create enough tension to rupture the tendon [17].

She et al. evaluated the rates of re-rupture in operative and non-operative management of TA injury. Patients with integrated functional rehabilitation programmes in their postoperative course were found to have significantly lower rates of re-rupture regardless of initial management. The highest re-rupture rates are in those who had non operative management followed by non-accelerated functional rehabilitation. By contrast, regardless of operative or non-operative management in the accelerated functional rehabilitation group the re-rupture rates are the same.

What is interesting from this study also is that of the eight studies that included ‘return to sport’ in their results the authors concluded that there was no difference in timeline between operative and non-operative approaches [18].

Research has also been conducted to compare open and percutaneous approaches to operative management with the theory behind the percutaneous approach being that less damage to the paratenon decreases the disruption to the blood supply to the TA and thus may help with repair. Percutaneous techniques offer the advantage of decreased infection rates but overall there is no significant difference again in terms of re-rupture, satisfaction or return to sport [19], [20]

Our accelerated Achilles tendon rehabilitation programme in MRHT is overseen by a consultant orthopaedic surgeon who specialises in foot and ankle surgery and is implemented by a dedicated multidisciplinary team of doctors, nurses, physiotherapists and occupational therapists.

The programme is split into the following:

**Phase 1**: Immediate Post Op/Recovery from surgery (Weeks 0–2)
**Phase 2**: Intermediate Post-Op (Weeks 3–6)

It is at this point that we transition the patient to a neutral position in the walking boot and out of boot active range of motion (neutral only in dorsiflexion)

**Phase 3:** Late Post-Op/Transitional (Weeks 7–12)

Gait retraining and gradual weaning out of the boot is encouraged at this point

**Phase 4:** Advanced Rehab Stage (Weeks 12–16)

Full active ROM encouraged aiming for 75% of functional strength of uninjured leg

**Phase 5**:Return to Competitive Sport (6–7 months)

Each phase as seen above, has dedicated aims, dressing specifications (if appropriate), movement requirements, and weightbearing status. Clinical criteria required to progress to the next phase are outlined clearly to facilitate patient progress seamlessly by allied health care professionals.

This particular regimen has been employed with great success over the last number of years.

It has been widely published in the literature that detraining and overall activity levels decreased across elite athletes, amateur athletes and the general population all over the world due to the COVID 19 pandemic [21], [22], [23], [24].

Although we focus on the middle aged ‘Weekend Warrior’ in this case series, it is to be expected that as restrictions ease and people resume normal activities we are likely to see increases in tendinous injuries across a spectrum of patient cohorts. This was seen on a professional level previously after the 2011 NFL Lockdown and more recently in the German Bundesliga [25], [26]. As such knowledge and implication of the accelerated rehabilitation programme for Achilles tendon rupture is both pertinent and topical.

In conclusion, this paper highlights the dangers inherent when well intentioned, but physically deconditioned individuals endeavour to perform a physical exercise which is deceptively demanding. Going forward, viral challenges such as the ‘Jerusalema’ may contribute to new and interesting mechanisms of injuries in our ‘weekend warrior’ cohort. Level one evidence suggests that conservative treatment is just as effective as surgical treatments in the majority of patients with an Achilles tendon rupture, as long as a protocol of rehabilitation with early weightbearing is performed. Our accelerated rehabilitation programme in MRHT is in line with others however internal audit and new literature in the future may enable us to refine it further.

## Key learning point

- An accelerated rehabilitation programme with early weightbearing is shown to have lower re-rupture rates that those treated more conservatively in patients with Achilles Tendon rupture.

## Ethical approval

N/A

## Funding

No sources of funding have been received for this manuscript.

## CRediT authorship contribution statement

Ryan Roopnarinesingh – Main author, conception of study design, assistant operator during surgery of one of the cases and followed patients up in the Outpatients department, revised manuscript and approval of version of manuscript for publishing

Robert Kenyon – Contributing author, conception of study design, revised manuscript and approval of version of manuscript for publishing

Luke Turley – Assistant surgeon in one of the cases, followed patient in the Outpatients department and monitored progress aided reviewing manuscript and approved for publishing

Aoife Feeley – Assistant surgeon in one of the cases, followed patient in the Outpatients department and monitored progress, aided reviewing manuscript and approved for publishing

Khalid Merghani – Consultant orthopaedic surgeon under whom care for 1× patient was provided. Operating surgeon. Conception of study design revised manuscript and approval of version of manuscript for publishing

Thomas Bayer - Consultant orthopaedic surgeon under whom care for 2× patient was provided. Operating surgeon. Conception of study design revised manuscript and approval of version of manuscript for publishing

## Guarantor

Dr. Ryan Roopnarinesingh

Senior House Officer

Midlands Regional Hospital Tullamore

Co.Offaly

Ireland

## Consent

Written informed consent was obtained from the patient for publication of this case report and accompanying images. A copy of the written consent is available for review by the Editor-in-Chief of this journal on request.

## Note

- The research work has been reported in line with the PROCESS criteria and SCARE Criteria [27].

## Provenance and peer review

Not commissioned, externally peer-reviewed.

## Declaration of competing interest

There are no conflicts of interest.
